# Determination of the Oxidative Stress Biomarkers of 8-Hydroxydeoxyguanosine and Dityrosine in the Gills, Skin, Dorsal Fin, and Liver Tissue of Atlantic Salmon (*Salmo salar*) Parr

**DOI:** 10.3390/toxics10090509

**Published:** 2022-08-29

**Authors:** Junjie Zhang, Eivind B. Sundfør, Rolf Klokkerengen, Susana V. Gonzalez, Vasco C. Mota, Carlo C. Lazado, Alexandros G. Asimakopoulos

**Affiliations:** 1 Department of Chemistry, Norwegian University of Science and Technology, Høgskoleringen 5, 7491 Trondheim, Norway; 2 Nofima, Norwegian Institute of Food, Fisheries and Aquaculture Research, 9019 Tromsø, Norway; 3 Nofima, Norwegian Institute of Food, Fisheries and Aquaculture Research, 1433 Ås, Norway

**Keywords:** oxidative stress, 8-hydroxydeoxyguanosine, dityrosine, Atlantic salmon, UPLC-MS/MS

## Abstract

Oxidative stress is a condition caused by an imbalance in the occurrence of reactive oxygen species in the cells and tissues of organisms. An ultra-performance liquid chromatography–electrospray ionization tandem mass spectrometry (UPLC–ESI–MS/MS) method was developed for the simultaneous determination of two oxidative stress biomarkers, 8-hydroxydeoxyguanosine (8OHDG) and dityrosine (DIY), in the gills, skin, dorsal fin, and liver tissue of Atlantic salmon (*Salmo salar*) parr. The use of target analyte-specific ^13^C and ^15^N internal standards allowed quantification of each target analyte to be performed through the standard solvent calibration curve. The relative recoveries [mean ± (relative standard deviation%)] of 8OHDG and DIY were 101 ± 11 and 104 ± 13% at a fortified concentration of 10 ng/mL (8OHDG) and 500 ng/mL (DIY), respectively, ensuring the accuracy of the extraction and quantification. The chromatographic separation was carried out using a gradient elution program with a total run time of 5 min. The limits of detection (LODs) were 0.11 and 1.37 ng/g wet weight (w.w.) for 8OHDG and DIY, respectively. To demonstrate the applicability of the developed method, it was applied in 907 tissue samples that were collected from Atlantic salmon parr individuals reared in an experimental land-based recirculating aquaculture system (RAS) treated with peracetic acid. Moreover, the possibility of using the dorsal fin as an alternative matrix for the minimally invasive assessment of oxidative stress in Atlantic salmon parr was introduced. To our knowledge, 8OHDG and DIY were used for the first time as biomarkers for biomonitoring the fish health (oxidative stress) of Atlantic salmon parr in RAS.

## 1. Introduction

Oxidative stress is a condition caused by an imbalance between the production and accumulation of reactive oxygen species (ROS) in cells and tissues and the inability of the biological systems to detoxify ROS [1]. ROS can cause damage to the DNA and proteins, consequently generating break-down chemical species [1,2,3,4,5]. In fish, the effects of oxidative stress are mainly assessed through the measurement of the “protein carbonyl content”, the ratio of the total antioxidant capacity (TAC) to the reactive oxygen and nitrogen species (ROS/RNS), lipid peroxidation, and the transcript and protein expression of antioxidants [6,7,8,9], while there are also reports of bioanalytical approaches for targeted analysis of specific biomarkers (e.g., metabolites) [10]. 8-Hydroxydeoxyguanosine (8OHDG) and dityrosine (DIY) are such specific biomarkers of DNA and protein damage from oxidative stress since their actual precursors are guanosine (a nucleoside consisting of guanine and ribose) and tyrosine (amino acid), respectively [2,3]. These biomarkers can also be used in the evaluation of drug toxicity in aquaculture [11,12]. The determination of 8OHDG is reported in fish tissues and its concentrations are associated with the exposure to specific xenobiotic substances [6]. For instance, the concentration of 8OHDG in the gill cells of freshwater Van fish (*Alburnus tarichi*) increased when exposed to trace concentrations of bisphenol A (contaminant; plasticizer) for 48 h, indicating peroxidation and genotoxicity effects [13]. Elevated concentrations of 8OHDG in the intestines of common carp (*Cyprinus carpio*) were also observed under increasing hydrogen peroxide (H_2_O_2_) exposure [14]. However, such a significant increase in 8OHDG concentrations was not documented in the other tissues (muscle, gills, and liver) of the common carp [14]. In contrast to the use of 8OHDG as a biomarker of oxidative DNA damage, DIY is rarely documented as a biomarker for oxidative protein damage in fish species [6,15]. Nonetheless, it is extensively documented in humans that DIY is a biomarker for oxidative protein damage [16], and it is used often as a tracer for specific human diseases [17,18,19].

Currently, ultra-performance liquid chromatography tandem mass spectrometry (UPLC–MS/MS) is used to determine 8OHDG and DIY concentrations in biological samples [16,20,21]. Apart from the chromatographic-based methods, commercialized ELISA (enzyme-linked immunosorbent assay) kits are available in the market for the sole determination of either 8OHDG or DIY. Nowadays, 8OHDG analysis in fish is mostly performed with these specific kits/antibody methods [13,22,23]. However, the selectivity of ELISA in trace concentrations can be compromised from cross-reactivity with other co-occurring analogs, while the simultaneous determination of 8OHDG and DIY with a single assay is, to the best of our knowledge, currently not available. Moreover, bioanalytical approaches are still lacking for the analysis of specific biological tissues such as the liver and gills [24].

With this as background, an extraction methodology tailored to UPLC–MS/MS analysis was developed in the present study for the simultaneous determination of 8OHDG and DIY. The method was applied in 907 tissue samples (skin, gills, dorsal fin, and liver) that were collected from Atlantic salmon parr individuals reared in an experimental recirculating aquaculture system (RAS) treated with peracetic acid (to test its effect on Atlantic salmon) [25]. It is noteworthy that guanosine and tyrosine concentrations are altered in the plasma of salmon smolts when exposed to peracetic acid, rendering 8OHDG and DIY promising biomarkers of oxidative stress [26,27,28]. Farmed Atlantic salmon is exposed to several factors that could trigger oxidative stress, including environmental parameters [29], water treatment [30], chemotherapeutics [31,32], and feed [33], among others. Thus, the objectives of this study were to (1) investigate the occurrence and concentration profiles of these two biomarkers in four selected fish tissues and (2) establish, where possible, associations of the biomarkers between the tissues. To our knowledge, 8OHDG and DIY are used for the first time as biomarkers for monitoring the fish health (oxidative stress) of Atlantic salmon parr in RAS.

## 2. Methods and Materials

### 2.1. Chemicals and Materials

8OHDG (≥98%) was purchased from Sigma–Aldrich (Darmstadt, Germany). DIY (≥98%) was purchased from MedChemExpress (Monmouth Junction, NJ, USA). 8-hydroxy-2′-deoxyguanosine-^15^N_5_ (^15^N_5_-8OHDG, 98%, 25 μg/mL) and O,O′-Dityrosine-^13^C_12_ (^13^C_12_-DIY, 99%) were purchased from Cambridge Isotope Laboratories, Inc. (Tewsbury, MA, USA). Methanol (MeOH; LC–MS grade) and water (LC–MS grade) were purchased from VWR Chemicals (Oslo, Norway). Ammonium formate (≥97%) was purchased from Sigma–Aldrich (Darmstadt, Germany). Acetic acid (analytical grade) was purchased from VWR Chemicals (Oslo, Norway). HybridSPE cartridges (HybridSPE-PhospholipidUltra, Supelco) and formic acid (≥98%) were purchased from Sigma–Aldrich (Darmsadt, Germany). The ACQUITY UPLC HSS T3 (100 × 2.1 mm 1.7 μm) and Kinetex C18 (30 × 2.1 mm, 1.3 µm) columns were purchased from Waters (Milford, MA, USA) and Phenomenex Inc. (Værløse, Denmark), respectively. An ULTRA C18 guard column (20 × 2.1 mm) was also purchased from Phenomenex Inc. (Værløse, Denmark).

Individual stock solutions of 100 μg/mL in MeOH were prepared for 8OHDG, DIY, and ^13^C_12_-DIY. The 100 μg/mL stock solutions of 8OHDG and DIY were used to prepare the working standard mixture solution of 10 μg/mL. The stock solutions of ^13^C_12_-DIY (100 μg/mL) and ^15^N_5_-8OHDG (25 μg/mL) were used to prepare the working internal standard mixture solution of 1000 ng/mL. Both working standard mixture solutions of the target analytes (TAs) and their internal standards (ISs) and their calibration standard solutions were prepared in MeOH/water (3:2, *v/v*). All standard solutions, except for ^15^N_5_-8OHDG (4 °C; darkness), were stored in the dark at −20 °C.

### 2.2. Sample Collection

The skin, gills, dorsal fin, and liver were collected from 234 Atlantic salmon parr (4 × 234 = 936 samples) at the Tromsø Aquaculture Research Station in Kårvik, Norway. The fish were 12.7 ± 1.5 cm long, weighed 29.3 ± 9.8 g (mean ± SD), and were maintained in a RAS during October–December 2020 [25]. Before sample collection, the fish were humanely euthanized with an overdose of Benzoak vet (ACD Pharmaceuticals AS, Leknes, Norway). Samples (ca 2 × 1 cm) were dissected: the skin was collected from the dorsal region immediately below the dorsal fin, the second gill arch was used for the gills, and the ventral side was used for the liver. All samples were placed in 1.5 mL polypropylene (PP) eppendorf tubes, snap frozen in dry ice, and transported to the NTNU analytical laboratory frozen. All activities in this study adhered to the guidelines and protocols concerning the ethical use of animals in research according to the European Union Directive 2010/63/EU and were approved by the Norwegian Food Safety Authority (FOTS ID 24128). Limitations for analysis were registered for 29 individual samples (e.g., due to a limited sample amount) out of the 936 samples (error 3%). Thus, *n* = 907 (skin: 222, gill: 232, dorsal fin: 225, liver: 228) tissue samples were eventually analyzed; all samples were stored in the dark at −20 °C until analysis. For method development/validation, a homogeneous fish pooled sample from all 4 matrices was prepared and stored under the same conditions as the actual samples.

### 2.3. Sample Preparation

Extraction protocol. The tissue samples were thawed at room temperature, and a portion of ~100 mg of each tissue sample was transferred into a 15 mL PP tube. Samples were fortified with 15 μL of 1000 ng/mL ISs followed by the addition of 600 μL MeOH containing 1% ammonium formate (*w/v*). Thereafter, the samples were vortex mixed (30 s) and ultrasonicated (30 min) followed by centrifugation (5 min, 3500 rpm; Centrifuge 5810, Eppendorf, Hamburg, Germany). The supernatant from each sample was collected and transferred into a new 15 mL PP tube.

Clean-up procedures. Two different purification methods were tailored to the extraction protocol.

In the first method (A), the extract was passed directly through a pre-washed (with 1 mL MeOH) HybridSPE cartridge (30 mg, 1 mL) and was collected in a vial. Then, 400 μL of water were added to the extract, and the vial was transferred for UPLC–MS/MS analysis.

In the second method (B), 400 μL of water were added to the obtained supernatant and kept in the freezer at (−20 °C) for 1 h to remove any formed lipid layer(s). After centrifugation (5 min, 10,000 rpm; Centrifuge 5415 D, Eppendorf, Hamburg, Germany), the supernatant (extract) was transferred for UPLC–MS/MS analysis.

### 2.4. Instrumental Analysis

The chromatographic separation was carried out using an Acquity UPLC I-Class system (Waters, Milford, CT, USA) coupled to a triple quadrupole mass analyzer (QqQ; Xevo TQ-S) with a ZSpray ESI ion source (Waters, Milford, CT, USA). The analytical column ACQUITY UPLC HSS T3 (100 × 2.1 mm, 1.7 μm) was connected to an ULTRA C18 guard column (20 × 2.1 mm). The mobile phase consisted of water with 0.1% (*v/v*) acetic acid (A) and methanol (B). The initial mobile phase composition was 100% A, held for 1 min, and then decreased to 15% A in 2.5 min, increased to 100% A in 0.5 min and held for another 1 min, for a total run time of 5 min. The flow rate was kept at 0.2 mL/min. The temperature in the autosampler was set at 10 °C. The injection volume was 2 µL, while the column temperature was set at 40 °C. Electrospray ionization under positive ionization mode (ESI+) was used for the analysis. The MS/MS detector parameters were set as follows: 150 °C for source temperature, 500 °C for desolvation temperature, 1000 L/h for desolvation gas flow, 150 L/h for cone gas flow, 0.15 mL/min for collision gas flow, 6 bar for nebulizer gas flow, and +2.5 kV for capillary voltage.

### 2.5. Method Validation

The calibration of the ESI method was verified by injecting solvent calibration standards at concentrations of 0.02–200 ng/mL (0.02, 0.05, 0.1, 0.2, 0.50, 1.00, 2.50, 5.00, 10.0, and 20 ng/mL for 8OHDG, and 0.10, 0.20, 0.50, 1.00, 2.00, 5.00, 10.0, 20.0, 50.0, 100, and 200 ng/mL for DIY). Precision was assessed through reproducibility experiments. For method reproducibility (method inter-day precision) experiments, the pool sample was fortified at the concentration of 500 ng/mL for DIY (since the pool sample contained a significant endogenous concentration) and 10 ng/mL for 8OHDG, and 18 replicate analyses (*n* = 18) were performed. For the instrumental repeatability (instrumental intra-day precision) experiments, the solvent matrix was fortified at a concentration of 10 ng/mL, and 18 replicate analyses (*n* = 18) were performed. The method limit of detection (LOD) and quantification (LOQ) was estimated for each TA (from the respective isotope-labelled internal standard) as 3 and 10 times the signal from the baseline noise (S/N ratio), respectively, in pool sample matrix. The accuracy (trueness) was evaluated through recovery experiments at the fortified concentrations of 1 (low level), 10 (medium level), and 100 (high level) ng/mL for 8OHDG and 50 (low level), 500 (medium level), and 5000 (high level) ng/mL for DIY; absolute and relative recoveries% (as defined in [34]) were calculated in three replicates (*n* = 3) at all three concentration levels. It is noteworthy that all samples fortified or determined with concentrations >20 ng/mL for 8OHDG and >200 ng/mL for DIY were diluted accordingly so that their instrumental response would fall in the linear range of the respective calibration curve for appropriate quantification. A calibration standard and a methanol solution were injected after every 20 samples as a check for drift in instrumental sensitivity and carryover effects of the TAs between samples, respectively.

### 2.6. Data Analysis

UPLC–MS/MS data were acquired with MassLynx v4.1 software, while quantification processing was performed with TargetLynx (Waters, Milford, MA, USA). Excel (Microsoft 2018, Washington, DC, USA), SPSS Statistics (IBM, version 27, Armonk, NY, USA), and GraphPad prism 8 (2019, San Diego, CA, USA) were used for general descriptive statistics. Data (concentration values) were log-transformed prior to performing Spearman correlation and principal component analysis (PCA). A t-test was used to test the significance of differences. The probability value of *p* < 0.05 was set for statistical significance. Values below the limits of detection (LODs) were substituted with a value equal to the LOD of the respective target analyte divided by a factor of √2. Concentrations were reported as ng/g wet weight (w.w.).

## 3. Results and Discussion

Method Development. The chromatographic retention of the TAs with the two tested chromatographic columns showed that the TAs exhibited poor retention on a classic C18 column (Figure S1a). This was attributed to the low molecular weight (M.W.), the high polarity of the TAs, and the distribution coefficient (Log P) of −1.32 and −0.31 for 8OHDG and DIY, respectively [35,36]. Optimal retention was achieved with the HSS T3 column, which even though it utilizes a C18 alkyl phase, it is bonded at a ligand density that promotes polar compound retention and demonstrates 100% aqueous mobile-phase compatibility. The retention times (RT; min) of 8OHDG and DIY with the HSS T3 column were 3.20 and 3.53 min, respectively (Figure S1b), while secondary chemical equilibria affected retention for both TAs with the C18 column. Martinez et al. (2018) used the Agilent Zorbax Aq column (2.1 × 150 mm, 3.5 µm) that has similar properties and function to the HSS T3 column, demonstrating retention times of 7.60 and 8.04 min for 8OHDG and DIY, respectively [16].

Moreover, the effects of different solvent mixture ratios of water and methanol on signal intensity were assessed (Figure S2). At a methanol/water ratio of 6:4 *v/v*, the signal intensity (peak height) of 8OHDG was the highest. The intensity of DIY decreased as the portion of water decreased; the highest intensity was obtained at the methanol/water ratio of 1:9 *v/v*, while at the optimal ratio of 8OHDG (methanol/water, 6:4 *v/v*), the intensity of DIY was decreased by ~25%. Eventually, a solvent composition of methanol/water (6:4 *v/v*) was chosen as a compromise for the in-vial solvent composition. The instrumental tandem mass spectrometry parameters of the TAs and ISs are listed in Table 1.

Extraction and purification. The absolute recoveries of the extraction and clean-up procedures for DIY and 8OHDG (method A and B) are presented in Table S1. The use of methanol created a cloudy suspension that was visible after the sample was extracted (method B), and the extract could not be obtained translucent post centrifugation. Thus, methanol was not further assessed. For DIY, the extraction efficiency of methanol solution containing 1% *w/v* ammonium formate (A) was optimal (based on the corresponding peak intensity; Figure S3). The methanol/water solvent mixture (1:1 *v/v*) (B) (A vs. B: *p* = 0.058, *t*-Test) demonstrated statistically significant lower performance (Figure S3), while the methanol solution containing 1% *v/v* formic acid (C) demonstrated the lowest performance (A vs. C: *p* = 0.002, *t*-Test) (Figure S3). Among those three tested solvents, the extraction efficiency for 8OHDG was not statistically different (*p* > 0.05, *t*-test). Therefore, a methanol solution containing 1% *w/v* ammonium formate was selected as the optimal extraction solvent for the method, and the effects of two consecutive extraction cycles in the extraction protocol were further assessed (method B). The absolute recoveries were presented as the mean ± (Relative Standard Deviation; RSD%; Table S1). The absolute recovery rates (%) of 8OHDG and DIY from the first extraction cycle were 59 ± 5.8% (*n* = 3) and 72 ± 4.6% (*n* = 3), respectively, and from the second extraction cycle they were 18 ± 1.9% and 25 ± 1.5%, respectively. The results indicated that two consecutive extraction cycles are optimal for quantitative recovery of the TAs. However, due to the need for a high-throughput analytical method, one extraction cycle was also deemed sufficient for our purpose due to the low uncertainties that were obtained (RSDs < 10%) and the use of TA-specific ISs.

**Figure 1:**
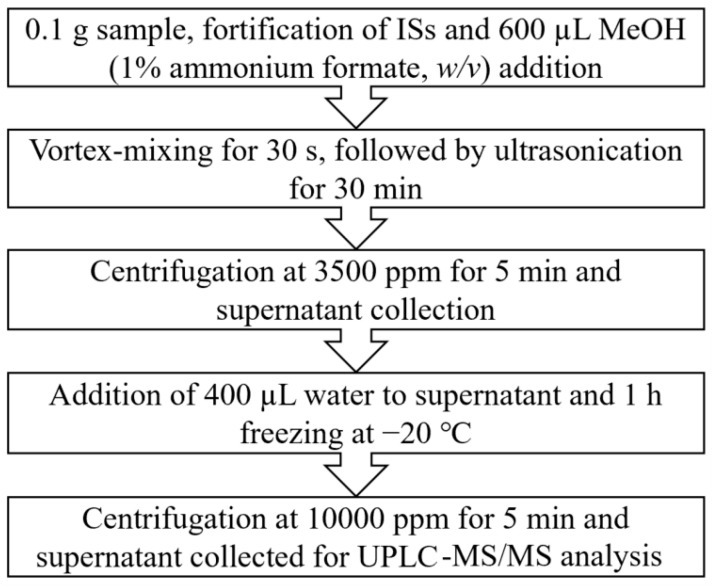
Sample preparation workflow.

The HybridSPE can be used for the purification of biological sample extracts [37,38,39], and it was found that the absolute recovery of 8OHDG was 23 ± 3.9% (relative recovery: 80 ± 24%), while DIY was detected in only one sample out of three replicates with an absolute recovery of 1.1% (relative recovery: 27%) (method A; Table S1). The reason for the low recovery of DIY is that it contains carboxyl (-COOH) groups, while the HybridSPE Zr-Si stationary phase co-retains such compounds with acidic functional groups along with the phospholipids [40]. Freezing (−20 °C) was applied for protein precipitation [41], and it was found that the response of each TA (the extracts derived from method B) was not statistically different (8OHDG: *p* = 0.967, DIY: *p* = 0.562; *t*-test; Figure S4) between the freeze and non-freeze treatment. However, a freezing step prior to sample injection was used to avoid protein buildup in the analytical instrument since it was visually evident that in the freeze-treated samples, a white precipitate formed post centrifugation (Figure S5). The final protocol is presented in Figure 1.

Method Performance. For the assessment of matrix effects, fortification was performed with 500 and 10 ng/mL for DIY and 8OHDG, respectively. The matrix effects (ME%; calculated by Equation (1)) of 8OHDG and DIY with the HybridSPE (method A) were −55 ± 5.7% and 4.0 ± 16.2%, respectively.

(1) $$\mathrm{ME}\%=\left(\frac{Peak\;area\;of\;post-extraction\;spiked\;matrix-Peak\;area\;of\;endogenous\;sample\;concentration}{Peak\;area\;of\;standard\;in\;pure\;solvent}-1\right)\times 100$$

Although DIY did not experience any matrix suppression, the absolute recoveries were not satisfactory (as mentioned above), and consequently, method A was used solely for confirmation purposes of samples with high DIY concentrations (>500 ng/g). The ion suppression of 8OHDG and DIY in method B was −81 ± 1.4% and −68 ± 0.5%, respectively, while the ion suppression in the second extraction cycle was found to be lower (8OHDG: −52 ± 1.3%, DIY: −45 ± 2.5%), indicating that most of the matrix interferences were already extracted in the first extraction.

**Figure 2:**
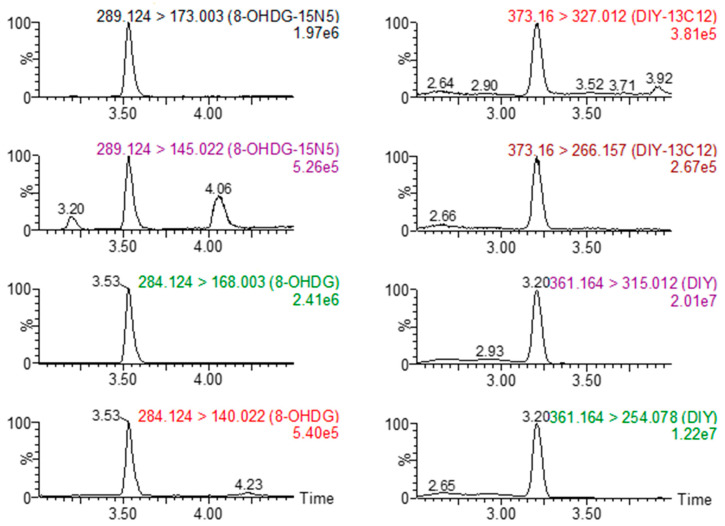
SRM chromatograms of 8OHDG, ^15^N_5_-8OHDG, DIY, and ^13^C_12_-DIY in a fortified fish liver sample.

**Figure 3:**
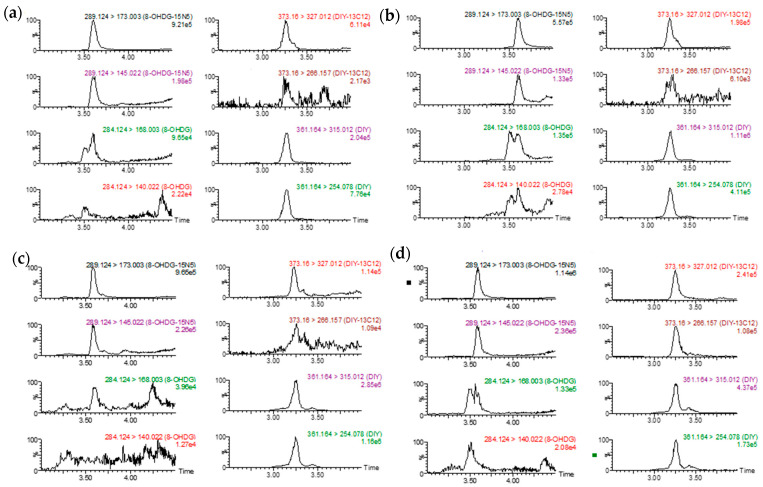
SRM chromatograms of TAs and ISs from randomly selected skin (**a**), gill (**b**), liver (**c**), and dorsal fin (**d**) samples.

The use of target analyte-specific ^15^N and ^13^C internal standard allowed for quantification to be performed through the standard solvent calibration curves and alleviated the need to perform quantification with matrix match curves. The relative recoveries of 8OHDG and DIY ranged from 96.3 to 114%, ensuring the accuracy of the extraction and quantification (Table S1). The instrumental correlation coefficients for both TAs were acceptable in the investigated intervals (r > 0.99). 8OHDG and DIY were not detected in the reagent blanks, which were prepared and analyzed as actual samples, denoting no background contamination whatsoever. Indicative Selective Reaction Monitoring (SRM) chromatograms of 8OHDG, ^15^N_5_-8OHDG, DIY, and ^13^C_12_-DIY from a fortified fish liver sample are presented in Figure 2, while indicative SRM chromatograms from random skin (a), gill (b), liver (c), and dorsal fin (d) samples are presented in Figure 3. The LODs for 8OHDG and DIY, which were both estimated as three times the signal-to-noise ratio of the instrument with a nominal wet sample mass of 100 mg (0.1 gr), were 0.11 and 1.37 ng/g, respectively (Table 2). The corresponding limit of quantification (LOQs) for 8OHDG and DIY, which were both estimated as ten times the signal-to-noise ratio of the instrument, were 0.37 and 4.57 ng/g, respectively. The inter-day precision of instrumental and method reproducibility was <10% (Table 2).

Method application. The determined concentrations for 8OHDG and DIY in the 907 samples analyzed are presented in Table 3. The detection rate% (DR%) of 8OHDG in 222 skin samples was 71%, and the concentrations ranged from <0.11 to 3.60 ng/g (median: 0.29 ng/g). DIY was found in 99% of the skin samples at a concentration range of <1.37 to 1518 ng/g (median: 167 ng/g). The concentration of 8OHDG in fish gill samples ranged from <0.11 to 914 ng/g (median: 4.70 ng/g), while DIY was found in 99% of the gill samples, ranging from <1.37 to 1278 ng/g (median: 417 ng/g). Among the 225 fish dorsal fin samples, 8OHDG was detected in 202 samples with concentrations ranging from <0.11 to 216 ng/g (median: 12.0 ng/g), while the detection rate of DIY was 78%, with concentrations ranging from <1.37 to 4050 ng/g (median: 343 ng/g). Among the 228 liver samples, 8OHDG was detected in 54 samples with concentrations ranging from <0.11 to 10.0 ng/g (median: <0.11 ng/g), while DIY was determined in most samples with a DR% of 97% and with a concentration ranging from <1.37 to 13176 ng/g (median: 3012 ng/g). Pre-extraction fortified pooled samples were used as QA/QC (Quality Assurance/Quality Control) samples and were prepared by spiking known concentrations of the TAs and ISs prior to extraction and clean-up. In total, 18 QA/QC samples were prepared by fortification of 10 ng/mL, and a method reproducibility (RSD%; *n* = 18) of 5.62 and 5.18% was documented for 8OHDG and DIY, respectively.

At present, mass spectrometry determination of 8OHDG and DIY is mostly focused on human urine samples [42,43,44,45]. For fish species, the analysis of 8OHDG in tissues (gills, muscle, liver, and intestines) is performed with enzyme-linked immunosorbent assays (ELISAs) [14,23] (Table 4). Most studies, except for one, reported the concentrations in units of ng/mg protein content [46], rendering it a challenge to establish an intercomparison in absolute numbers with the 8OHDG concentrations found in our study. To the best of our knowledge, there are no previous studies that reported DIY concentrations in fish tissues, while this is the first time that 8OHDG and DIY were detected in fish fin tissue.

**Figure 4:**
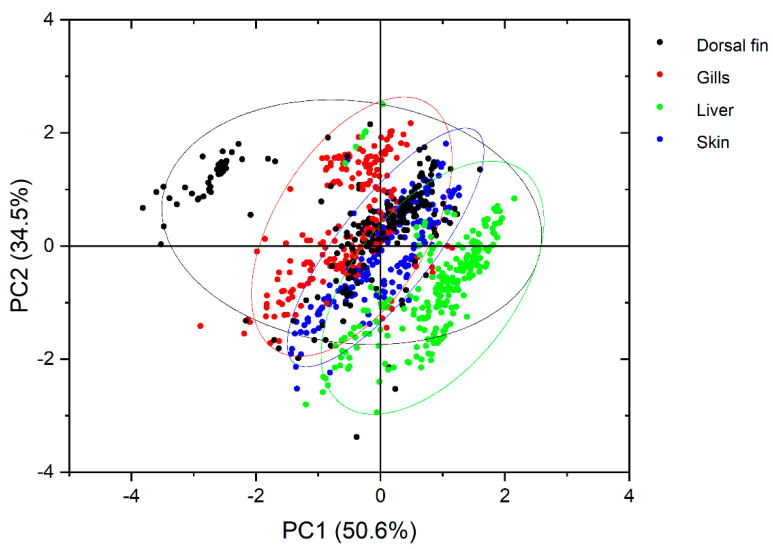
Principal component analysis (PCA) biplot of the classification of matrices based on the log-transformed concentrations (ng/g) of DIY and 8OHDG.

Through PCA, it was documented that there were significant differences in the concentration profiles of 8OHDG and DIY between the four tissues (Figure 4). Through Pearson correlation analysis (Table S2), it was found that the DIY concentrations in the skin and gills were significantly positively correlated (*r* = 0.338, *p* < 0.01), while the DIY concentrations in the dorsal fin were negatively correlated with those found in the skin (*r* = −0.287, *p* < 0.01) and gills (*r* = −0.224, *p* < 0.01). There was no correlation between the DIY concentrations in these three tissues with those in the liver. Only 8OHDG concentrations demonstrated a significant negative correlation between skin and liver (*r* = −0.856, *p* < 0.01), and no correlation was observed between the 8OHDG concentrations in the remaining tissues. It is noteworthy to report that the gills, skin, and dorsal fin are in direct contact with the external environment of the fish in contrast to the liver, and by-default, distinctly different concentration profiles among external and internal organs were expected. The skin and gills of salmon are very sensitive to exogenously generated oxygen radicals and have the robust capability to mount strong oxidative stress response by the mobilization of antioxidants [28,32]. The results introduced the possibility of using clipping of the dorsal fin as an alternative and minimally invasive approach for measuring the two biomarkers of oxidative stress in Atlantic salmon parr; fish fins have the capacity for regeneration after clipping [47]. Such differences across tissues were also previously documented for other oxidative stress biomarkers in salmon [32,48] and other fish species. Pandey et al. (2003) assessed oxidative stress through the monitoring of the glutathione reductase activity in the liver, stomach, and gills of the Indian freshwater fish (*Wallago attu*) across two distinctly located sites of the Yamuna River (India) that presented differences in their pollution loads [10]. Significant differences were documented between the two locations in the activity of glutathione reductase in the liver and gills of the fish, but no differences were observed in the stomach tissue samples [10]. In a recent study on farmed salmon, where the effects of peracetic acid were assessed, similar findings were observed; downregulation of selected genes occurred in the gills, upregulation occurred in the skin, and no substantial changes were observed in the liver [48].

## 4. Conclusions

A methodology tailored to UPLC–MS/MS was developed for the determination of two oxidative stress biomarkers, 8OHDG and DIY, in the gill, skin, dorsal fin, and liver tissue of Atlantic salmon parr. Target analyte-specific ^15^N_5_-8OHDG and ^13^C_12_-DIY internal standards were used for the effective compensation of matrix effects and extraction losses during analysis. This allowed for quantification to be performed with the standard solvent calibration curves and alleviated the need to perform it with matrix match curves in actual tissue samples. The method was used successfully in 907 fish tissue samples from Atlantic salmon, and both biomarkers were detected in most samples. The occurrence and concentration profiles of these two biomarkers were established in the examined tissues. Moreover, correlations of the biomarkers across tissues were uncovered, introducing the possibility of using the dorsal fin as an alternative matrix for the minimally invasive assessment of oxidate stress in Atlantic salmon parr. To our knowledge, 8OHDG and DIY are used for the first time as bioindicators for monitoring the oxidative stress status of Atlantic salmon parr in RAS.

## Figures and Tables

**Table 1:** UPLC–MS/MS parameters.

Compound	Retention Time	Precursor Ion	Quantification/Confirmation	Cone Voltage (V)	Collision Energy (ev)
8OHDG	3.53	284.1	140.0/168.0	22	30/12
DIY	3.20	361.1	315.0/254.0	18	24/22
^15^N_5_-8OHDG	3.53	289.1	145.0/173.0	22	30/12
^13^C_12_-DIY	3.20	373.1	327.0/266.1	18	24/22

**Table 2:** Calibration curves, limits of detection (LODs) and quantification (LOQs), and precision for the determination of 8OHDG and DIY.

	Calibration Range	r^2^	LOD (ng/g)	LOQ (ng/g)	Method Inter-day Precision ^a^ (%)	Instrumental Intra-Day Precision (%) ^a^
8-OHDG	0.02–20	0.99	0.11	0.37	5.62	3.21
DIY	0.1–200	0.99	1.37	4.57	5.18	2.49

^a^ Fortified concentrations: 10 ng/mL for 8OHDG and 500 ng/mL for DIY.

**Table 3:** Concentrations of 8OHDG and DIY in skin, gill, dorsal fin, and liver tissue samples from Atlantic salmon parr.

ng/g w.w.	Skin (*n* = 222)		Gills (*n* = 232)		Dorsal Fin (*n* = 225)		Liver (*n* = 228)	
	8OHDG	DIY	8OHDG	DIY	8OHDG	DIY	8OHDG	DIY
Max	3.60	1518	914	1278	216	4050	10.0	13176
Min	<0.11	<1.37	<0.11	<1.37	<0.11	<1.37	<0.11	<1.37
Mean ± st.dev.	0.33 ± 0.37	197 ± 128	25 ± 75	468 ± 202	7.5 ± 23	402 ± 507	0.41 ± 0.97	3534 ± 2175
25th	<0.11	128	1.41	329	0.48	196	<0.11	2020
Median	0.29	167	4.70	417	1.20	343	<0.11	3012
75th	0.42	229	18	568	3.2	498	<0.11	4704
DR% *	71	99	95	99	90	78	24	97

* DR%: Detection rate%.

**Table 4:** Reported concentrations of 8OHDG in fish tissues.

Compound	Fish	Sample	Analytical Technique	Concentrations	Ref.
8OHDG	*Alburnus tarichi Güldenstädt*	Gills	Antibody	25–37 ng/mL	[11]
8OHDG	*Cyprinus carpio*	Liver	ELISA	3.5–5.6 ng/mg prot	[12]
		Gills		5.5–7.5 ng/mg prot	
		Muscle		6.0–9.5 ng/mg prot	
		Intestines		2.8–5.2 ng/mg prot	
8OHDG	*Cyprinus carpio*	Gills	ELISA	4.8–6.6 ng/mg prot	[44]
8OHDG	*Oncorhynchus mykiss*	Liver	ELISA	0.45–1.05 ng/mg prot	[21]
8OHDG	*Salmo salar*	Liver	UPLC–MS/MS	<0.11–10 ng/g	This study
		Gills		<0.11–914 ng/g	
		Skin		<0.11–3.6 ng.g	
		Dorsal fin		<0.11–216 ng/g	

## Data Availability

All data are described in the manuscript and supplementary materials.

## Supplementary Materials

The following supporting information can be downloaded at: https://www.mdpi.com/article/10.3390/toxics10090509/s1, Figure S1: Total ion current chromatogram of 8OHDG and DIY separated by Kinetex C18 (30 × 2.1 mm, 1.3 µm) (a) and ACQUITY UPLC HSS T3 (100 × 2.1 mm, 1.7 μm) (b) at a concentration of 10 ng/mL for DIY and 8OHDG in solvent matrix; Figure S2. The intensity of 8OHDG and DIY in different solvent (M: MeOH; W: water; e.g., M_W_1_9 denotes methanol/water, 1:9 *v/v*) at a fortified amount of 10 ng for DIY and 8OHDG in solvent matrix; Figure S3. The intensity of 8OHDG and DIY in different extracted solutions (A: MeOH with 1% *w/v* ammonium formate; B: MeOH/Water (1:1 *v/v*); C: MeOH with 1% *v/v* formic acid) at a fortification level of 10 ng for DIY and 8OHDG in pooled matrix sample; Figure S4. The intensity of ^15^N_5_-8OHDG (a) and ^13^C_6_-DIY (b) in the samples with freeze and non-freeze treatment; Figure S5. The difference of the same sample treated with freeze and non-freeze treatment; Table S1: Absolute (AR; *N*=3 replicates) and relative recoveries (RR; *N*=3 replicates) of 8OHDG and DIY (Mean ± RSD %); Table S2. Spearman correlations of 8OHDG and DIY concentrations between the 4 tissues.
