# Supplementary material for: Determination of the Oxidative Stress Biomarkers of 8-Hydroxydeoxyguanosine and Dityrosine in the Gills, Skin, Dorsal Fin, and Liver Tissue of Atlantic Salmon (Salmo salar) Parr
Source: Toxics. 2022 Aug 29;10(9):509. doi: 10.3390/toxics10090509 (PMC9503732; doi:10.3390/toxics10090509)
Supplement: Supplementary file 1 [file toxics-10-00509-s001.zip › toxics-1864956-supplementary.pdf]

toxics MDPI

Supplementary Materials: Determination of the Oxidative Stress Biomarkers of 8-Hydroxydeoxyguanosine and Dityrosine in the Gills, Skin, Dorsal Fin, and Liver Tissue of Atlantic Salmon (*Salmo salar*) Parr

Junjie Zhang, Eivind B. Sundfør, Rolf Klokkerengen, Susana V. Gonzalez, Vasco C. Mota, Carlo C. Lazado and Alexandros G. Asimakopoulos

Table S1. Absolute (AR; N=3 replicates) and relative recoveries (RR; N=3 replicates) of 8OHDG and DIY (Mean ± RSD %).

	Method A				Method B			
	AR		RR		AR		RR	
	8OHDG	DIY	8OHDG	DIY	8OHDG	DIY	8OHDG	DIY
First extraction[a]	23±3.9	1.10*	80±24	27*	59±5.8	72±4.6	101±11	104±13
Second extraction[a]					18±1.9	25±1.5	100±10	114±12
Low[b]					71±7.2	71±5.7	101±4.7	96.3±1.8
High[b]					72±5.1	78±7.8	97±3.1	104±1.5

*Detected in one sample out of the 3 replicates.

[a]These two extractions were based on the medium fortified concentration (10 ng/mL for 8OHDG and 500 ng/mL for DIY)

[b]Low and High represent low (1 ng/mL for 8OHDG and 50 ng/mL for DIY) and high fortified concentrations (100 ng/mL for 8OHDG and 5000 ng/mL for DIY), respectively, and were performed with Method B.

Note: the final extraction protocol is highlighted with green color (method B). The second extraction cycle was not incorporated in method B (see main manuscript).

Table S2. Spearman correlations of 8OHDG and DIY concentrations between the 4 tissues.

DIY	skin	gills	dorsal fin	liver
skin		0.338**	−0.287**	−0.030
gills			−0.224**	0.039
dorsal fin				0.044
liver				
8OHDG	skin	gills	dorsal fin	liver
skin		0.079	0.138	−0.856**
gills			−0.177	−0.174
dorsal fin				0.265
liver				

**: $p<0.01$

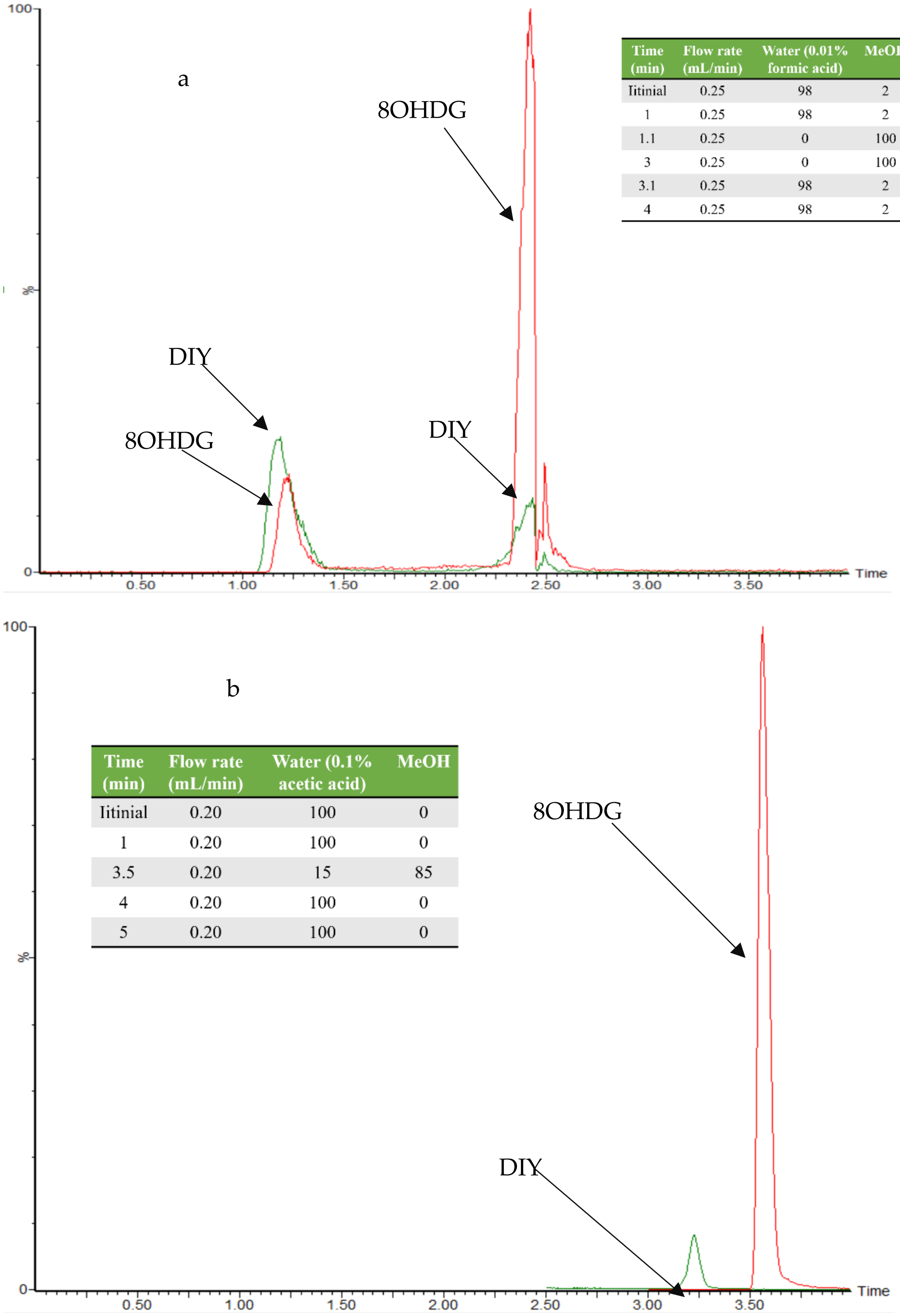

Time (min)	Flow rate (mL/min)	Water (0.01% formic acid)	MeOH
Iitinial	0.25	98	2
1	0.25	98	2
1.1	0.25	0	100
3	0.25	0	100
3.1	0.25	98	2
4	0.25	98	2

Time (min)	Flow rate (mL/min)	Water (0.1% acetic acid)	MeOH
Iitinial	0.20	100	0
1	0.20	100	0
3.5	0.20	15	85
4	0.20	100	0
5	0.20	100	0

Figure S1. Total ion current chromatogram of 8OHDG and DIY separated by Kinetex C18 (30 × 2.1 mm, 1.3 µm) (**a**) and ACQUITY UPLC HSS T3 (100 × 2.1 mm, 1.7 µm) (**b**) at a concentration of 10 ng/mL for DIY and 8OHDG in solvent matrix.

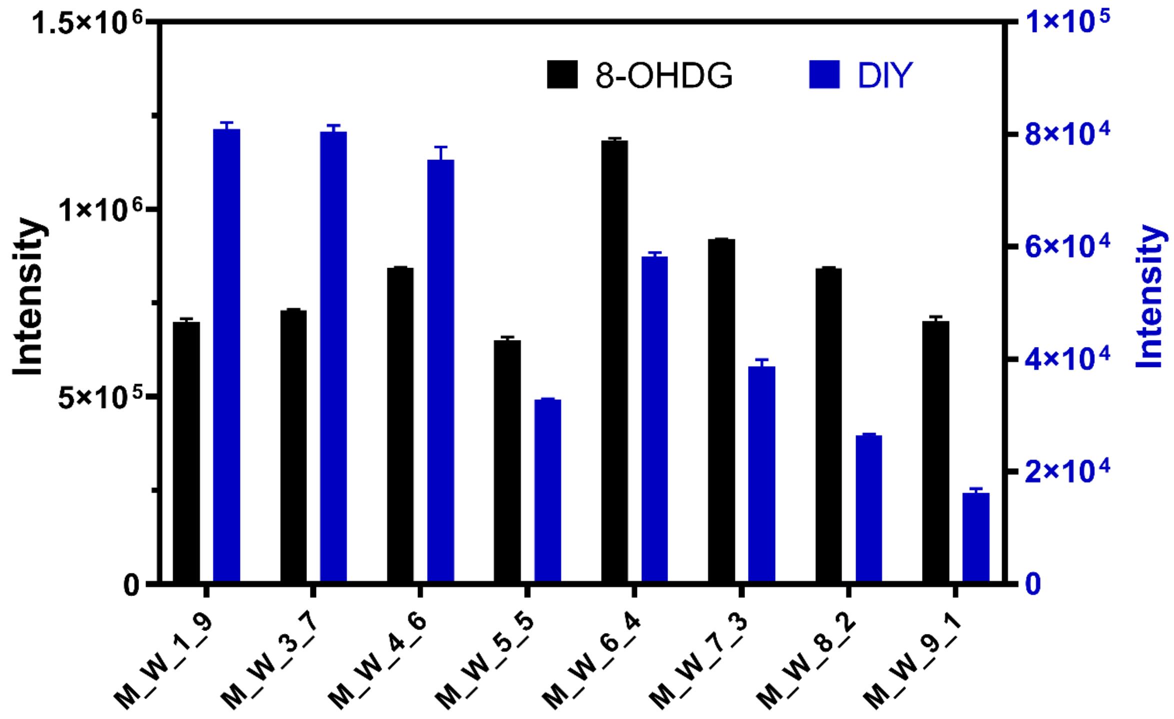

Figure S2. The intensity of 8OHDG and DIY in different solvent (M: MeOH; W: water; e.g., M_W_1_9 denotes methanol/water, 1:9 v/v) at a fortified amount of 10 ng for DIY and 8OHDG in solvent matrix.

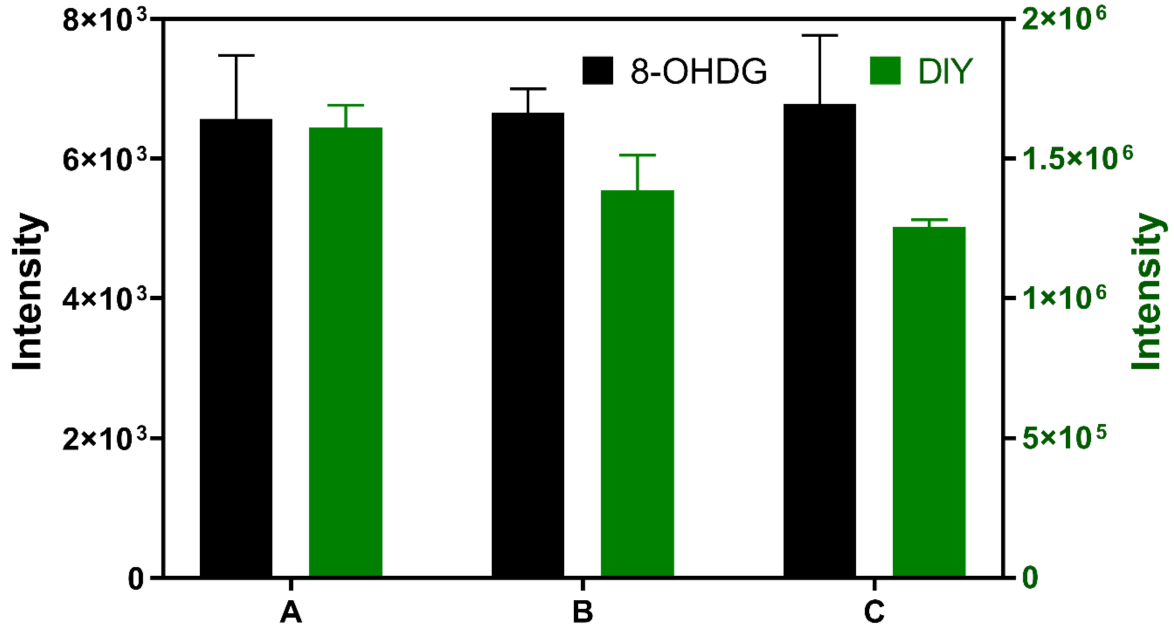

Figure S3. The intensity of 8OHDG and DIY in different extracted solutions (A: MeOH with 1% w/v ammonium formate; B: MeOH/Water (1:1 v/v); C: MeOH with 1% v/v formic acid) at a fortification level of 10 ng for DIY and 8OHDG in pooled matrix sample.

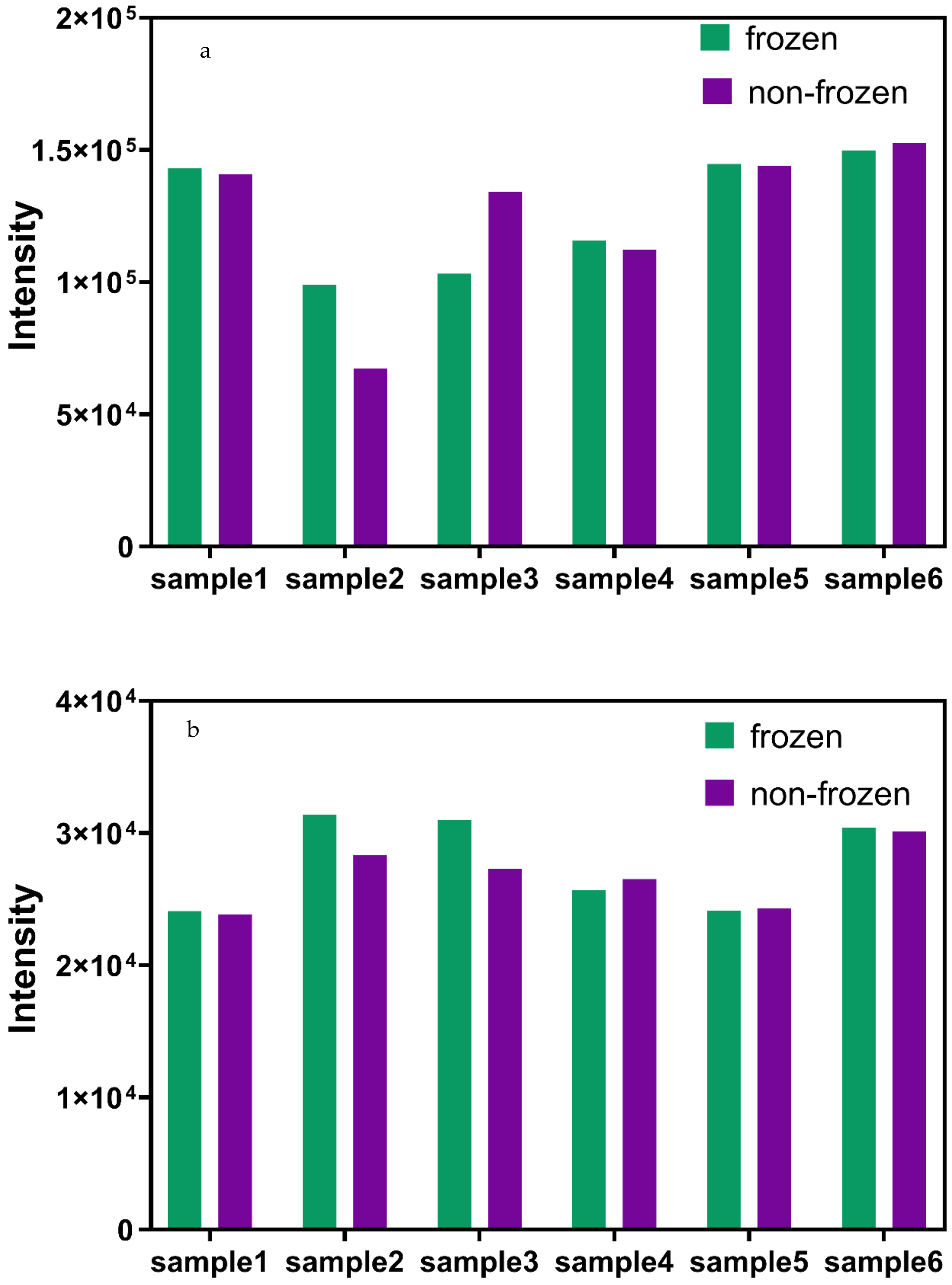

Figure S4. The intensity of $^{15}N_5$-8OHDG (a) and $^{13}C_6$-DIY (b) in the samples with freeze and non-freeze treatment.

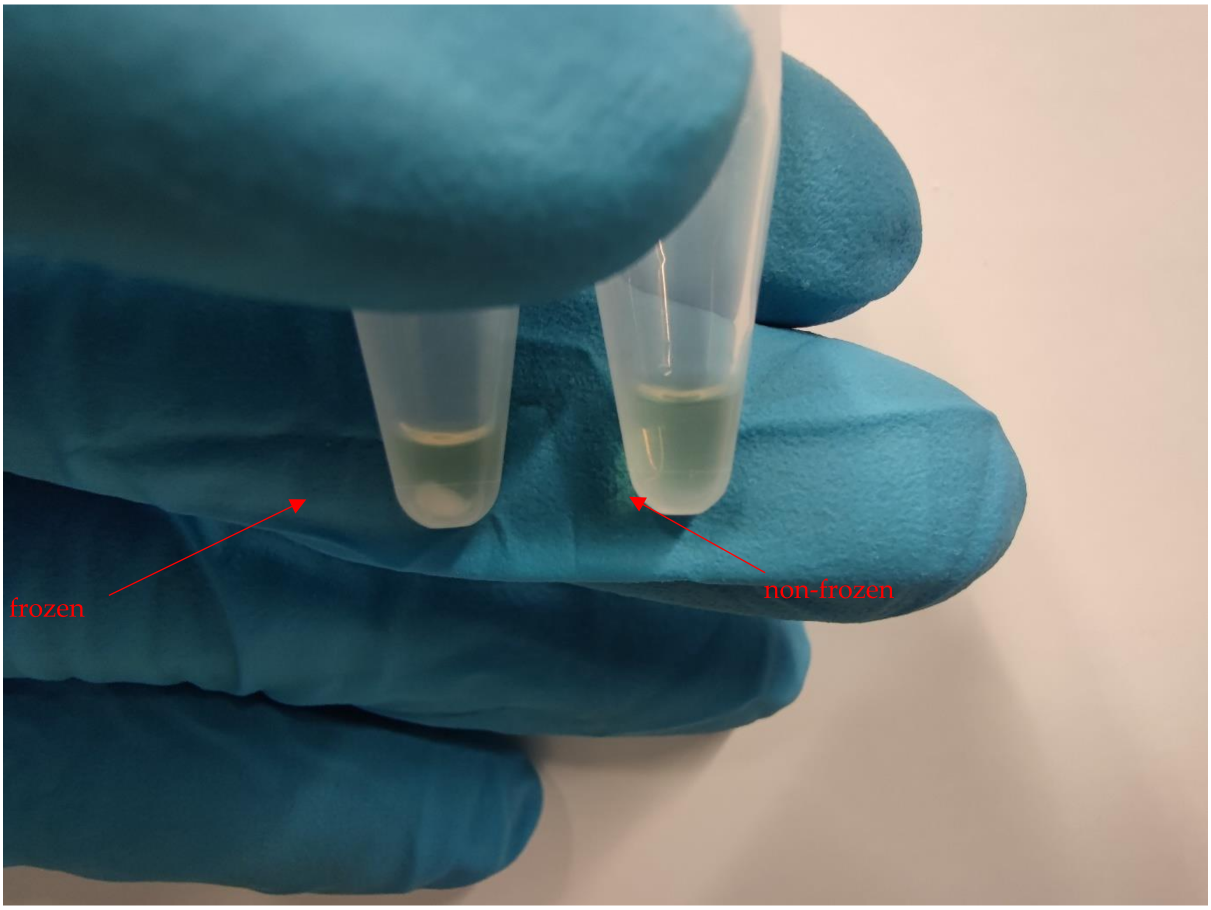

Figure S5. The difference of the same sample treated with freeze and non-freeze treatment.
